# Predictor of smoking cessation among school-going adolescents in Indonesia: a secondary analysis based on the transtheoretical model of behavioral change

**DOI:** 10.3389/fpsyt.2024.1374731

**Published:** 2024-03-07

**Authors:** Omid Dadras

**Affiliations:** ^1^ Department of Global Public Health and Primary Care, University of Bergen, Bergen, Norway; ^2^ Bergen Addiction Research, Department of Addiction Medicine, Haukeland University Hospital, Bergen, Norway

**Keywords:** tobacco smoking, adolescents, Indonesia, cigarette, predictor

## Abstract

**Introduction:**

This study elucidates the complex journey of adolescents toward smoking cessation, investigating the association of relevant demographic factors, advertising, promotion, anti-cigarette messages, and individual knowledge and attitudes with being in different smoking cessation stages.

**Methods:**

Utilizing data from the 2019 Indonesia Global Youth Tobacco Survey, this secondary analysis included adolescents who reported ever smoking. The Transtheoretical Model (TTM) guided the categorization of the outcome variable into three smoking cessation stages based on the responses to two questions related to the intention and timing of the smoking cessation. This included contemplation, action, and maintenance stages. Multinomial logistic regression analyzed the associations between each independent variable and being in each stage of smoking cessation. The study comprised 3596 Indonesian adolescents from grades 7-12, of which 2484 responded to two questions related to intention and timing of smoking cessation and were included in regression analysis.

**Results:**

Findings indicate that males and those aged ≥16 were predominantly in contemplation phase. Early smoking initiation, usage of other tobacco products, and exposure to various forms of smoke increased the likelihood of being in contemplation and action phases. Parental smoking, school smoking exposure, and second-hand smoke were significant contemplation phase predictors. Exposure to tobacco advertising was linked to an increased likelihood of being in contemplation and action phases, whereas anti-cigarette messages showed no significant impact. Awareness of cigarette and second-hand smoke harms reduced the odds of being in the contemplation phase, while enjoying smoking and willingness to accept cigarettes from friends increased the odds of being in contemplation and action phases rather than in maintenance phase.

**Conclusion:**

Addressing age, gender, cultural influences, environmental factors, and attitudes towards smoking through tailored interventions is vital for aiding smoking cessation in Indonesian adolescents. Strengthened tobacco control in schools and public places is recommended to bolster these efforts. Longitudinal studies are required to explore the evolving patterns of smoking cessation behaviors over time, enhancing our understanding of the factors influencing sustained cessation.

## Introduction

Tobacco use is a global public health concern, with over 1.3 billion people, accounting for 22.3% of the world population, using tobacco products in 2020 (1). The reports indicate a significantly higher prevalence in men (36.7%) than in women (7.8%) (1). In Southeast Asia, nearly 600 million smokers live within the region, with Indonesia having the third-highest number of tobacco users in the world (2). The impact of tobacco use is not limited to adults; it also affects younger generations. In 2022, more than 1 in 10 middle and high school students, approximately 3.08 million, had used a tobacco product during the past 30 days. This includes 16.5% of high school and 4.5% of middle school students (3). In Indonesia, the prevalence of smoking among Indonesian adolescents has been increasing during the last decades, with 19.2% of adolescents aged 13-15 reported ever using tobacco products in 2019 (4). Furthermore, 43.2% of Indonesian youths started to consume cigarettes when they were 12 to 13 years old (4). Adolescent smoking is a global epidemic, with over 50% of tobacco users being young people worldwide (5). The United States Department of Health and Human Services (USDHHS) has confirmed that teen smokers are at a higher risk of becoming addicted to nicotine and suffering from tobacco-related diseases such as respiratory and cardiovascular system damage (5). Thus immediate actions to address this issue are due and studies exploring the underlying drivers of such trends among adolescents are warranted.

Cigarette smoking among Indonesian adolescents is a pressing public health issue, with a variety of factors influencing their decision to smoke. Studies have identified that having friends who are addicted to smoking, being offered cigarettes, and having easy access to tobacco products are major contributors to the initiation of smoking in this demographic (6). Furthermore, the adolescent social environment plays a significant role, with peers exerting a major influence on changes in smoking behavior (7). Intergenerational smoking habits, where parental smoking behavior influences the offspring’s attitudes and behaviors toward smoking, also contribute significantly to this issue (7). Despite various interventions, the prevalence remains high, necessitating innovative approaches to address this issue. The lack of implementation and enforcement of tobacco advertising bans and the general public’s acceptance of tobacco advertising in Indonesia hampers effective tobacco prevention among adolescents (5). Addressing this problem requires a comprehensive understanding of these influencing factors and the development of targeted interventions that address the social and environmental factors contributing to adolescent smoking in Indonesia.

A noticeable gap in the existing literature is the lack of comprehensive analysis of factors that are associated with the stages of behavioral change, based on the Transtheoretical Model (TTM), related to smoking cessation among Indonesian adolescents. Despite the high prevalence of smoking among Indonesian adolescents, there is a lack of research on the factors that affect their decision to take action, quit smoking cigarettes, and maintain a smoke free life. The TTM is a theoretical framework that categorizes the stages of behavioral changes into five stages: pre-contemplation, contemplation, preparation, action, and maintenance (8). It offers a structured understanding of the internal and external factors influencing behavior change, making it apt for this study. Leveraging TTM, we categorized the smoking cessation behavioral changes into three phases including contemplation, action, and maintenance based on the available data from Indonesia Global Youth Tobacco Survey (GTYS) in 2019. We aimed to provide a nuanced understanding of the adolescents’ journey towards smoking cessation, examining the relationship of different demographic factors, exposure to advertisement, promotion, and anti-cigarette messages, as well as participants’ knowledge and attitudes with being in each stage of the process.

## Materials and methods

### Study setting

The Global Youth Tobacco Survey (GYTS), a component of the Global Tobacco Surveillance System (GTSS), is a global standard for systematically monitoring youth tobacco use (smoking and smokeless) and tracking key tobacco control indicators. In Indonesia, GYTS was conducted in 2019 by the National Health Research and Development (NHRD) under the Ministry of Health.

### Participants and sampling

The Indonesian GYTS 2019 was a cross-sectional study undertaken in Indonesia’s public and private schools to evaluate tobacco use among students aged 13 to 15 years old. The sample was categorized into three main regions: Java, Sumatra, and others. Each region comprises 25 junior high schools and 25 high schools, amounting to a total of 150 schools spread across 30 provinces. Sampling was carried out in two distinct stages: initially, schools were chosen based on a probability proportionate to their size (PPS), followed by a random selection of classes from various schools in the second stage. The entire students from the selected classes were eligible to participate in the survey (9). The overall response rate was 91.0%. A total of 9,992 eligible students in grades 7- 12 completed the survey, of which 5,125 were aged 13-15 years. However; in this study, we included all the adolescents who participated in the survey who ever smoked (n=3596). However, of 3596 students who reported ever smoked, only 2484 students responded to both questions related to intention and timing of the smoking cessations that inform the construction of the outcome variable and therefore, included in the regression analysis.

### Data collection

The interviewers initiated the data collection through a brief dialogue with the administrative authorities at the school, clarifying the specific class chosen for the study, with all its students capable of participating in the survey. The interviewing process is set to engage each student in the selected class and is estimated to last for 45 minutes. The research team has provided all necessary survey materials including questionnaires, answer sheets, and writing utensils for the students. Before distributing the questionnaires to the students, the interviewers provided clear instructions on how to properly complete the answer sheets and the questionnaire. All students and guardians were then provided with a consent form before the survey.

### Variables

#### Dependent variable

The TTM theory is chosen to specify the smoking cessation stages in which there are five stages to behavioral changes; namely pre-contemplation (not thinking to stop smoking), contemplation (thinking to stop smoking in the next 6 months), preparation (thinking to stop smoking within the next 30 days), action (has stopped smoking in 6 months), and maintenance (has stopped smoking for more than 6 months) (8). We have used the responses to two questions “Do you want to stop smoking now?”, representing the intention, and “How long ago did you stop smoking”, representing the timing, to construct the smoking cessation variable including three categories coded as “0” contemplation (thinking to stop smoking but not stopped yet), “1” action (has stopped smoking for last 3 months), “3” and maintenance (has stopped smoking for more than 3 months). The choice of 3 months for defining the action and maintenance stages was shaped by the choices to corresponding questions in GYTS 2019.

#### Independent variables

The independent variables in this study were classified into three categories including *individual factors*, *environmental factors*, and *knowledge and attitude*. Details of selected variables and corresponding questions and alternative responses were provided in Appendix 1.

##### Individual factors

Age (<14, 14-16, >16), sex (male, female), pocket money (Indonesian Rupiah), age at initiation of cigarette smoking (<10, 10-13, >13), ever tried other tobacco products (such as chewing tobacco, betel leaf with cane, and betel nut with tobacco), ever tried Shisha, ever tried smokeless tobacco products, and ever tried e-cigarette.

##### Environmental factors

Parental smoking; School environment (witness someone smoking inside the school in last 30 days, witness someone smoking inside the school in last 30 days, ever witness teacher smoking in school); Second-hand tobacco exposure (exposure to second-hand smoke inside the home, and at indoor or outdoor public places in last 7 days); advertising and promotions (have seen cigarette ads on TV or social media, at events or sale centers in last 30 days, has got free/discounted cigarettes from cigarette companies, can buy of cigarettes near school); anti-cigarette messages (have seen or heard anti-cigarette messages on social media or events in last 30 days). All variables were coded as 0 “no” and 1 “yes”.

##### knowledge and attitude

Awareness of cigarette or second-hand smoke harm; taught about tobacco harms in school in last year; cigarette smoking is joyful; willingness to use a cigarette if offered by a friend; support for smoking bans in outdoor or indoor public places.

### Statistical analysis

Descriptive statistics were employed to describe the distribution of individual and environmental factors as well as knowledge and attitude toward cigarette smoking among Indonesian adolescents in grades 7–12 who reported ever smoking. Multinomial Logistic regression analysis was used to examine the association between independent variables with being in each smoking cessation stage. Prior to regression analysis, adherence to the proportional odds assumption, and ensuring an adequate sample size for each category of the dependent variable was assessed. The results were reported as relative risk ratio (RRR) and 95% confidence interval (95%CI). Age and sex, as the most influencing factors on smoking cessation behavior (10, 11), were accounted for in all multivariate analyses to allow us to measure the independent effect of each factor on being in each smoking cessation stage, regardless of the potential confounding effects of age and sex (9). This adjustment also aimed to mitigate bias introduced by missing data in responses to questions regarding intention and timing of smoking cessation, which inform the study outcome. Understanding the independent association of each independent variable with being in each smoking cessation stage will help in formulating targeted interventions and preventive strategies at the school level for all students in grades 7–12 while providing valuable policy insight (10). Due to the complex sampling design in GYTS 2019, sampling design and weights were defined and applied in all analyses in STATA 17. The statistical significance level was set at p< 0.05.

## Results

In the GYTS 2019, a total of 3,596 Indonesian adolescents in grades 7-12 reported ever smoking. Among them, 2,484 responded to two questions regarding their intention and timing of smoking cessation. Of these respondents, 18.89% were in the contemplation stage, 19.73% in the action stage, and 61.38% in the maintenance phase. The following individual, environmental, and knowledge, and attitude factors were associated with being in each stage of smoking cessation:

### Individual factors influencing the smoking cessation behavior

#### Age

**Table 1** describes the associated individual factors with smoking cessation behaviors among school-going Indonesian adolescents in grades 7-12th. The significant factors are listed below. Approximately 33.78% of participants were below 14 years, 51.67% were between 14 and 16 years, and 14.54% were above 16 years. Compared to the maintenance phase, adolescents aged 14-16 years showed a higher likelihood of being in the contemplation phase with an RRR of 2.29 (95% CI: 1.68, 3.11). However, this age group did not show a significant with being in the action phase (RRR: 0.90, 95% CI: 0.71, 1.14). Adolescents older than 16 years exhibited a markedly higher propensity to be in the contemplation phase (RRR: 5.50, 95% CI: 3.78, 8.02), but again, no significant difference was observed for being in the action phase.

**Table 1:** Individual factors influencing the behavioral changes in smoking cessation among school-going Indonesian adolescents in grades 7-12, GYTS 2019.

		Contemplation vs. maintenance	Action vs. maintenance
	N (%)	RRR (95%CI)	RRR (95%CI)
Age (years)			
<14	1126 (33.78)	–	–
14-16	1817 (51.67)	2.29 (1.68, 3.11)	0.90 (0.71, 1.14)
>16	653 (14.54)	5.50 (3.78, 8.02)	0.98 (0.67-1.44)
Sex			
Female	649 (15.14)	–	–
Male	2955 (84.86)	3.77 (1.72-8.26)	3.17 (2.16-4.66)
Age at cigarette initiation			
<10	758 (21.71)	–	–
10-13	1828 (56.98)	1.41 (1.01, 1.97)	2.13 (1.40, 3.25)
>13	805 (21.30)	4.63 (3.06, 7.01)	2.97 (1.91, 4.62)
Pocket money (rupiah) ^1^			
<20000	1725 (48.05)	–	–
20000-40000	673 (18.67)	0.89 (0.70, 1.23)	0.96 (0.71, 1.28)
>50000	1200 (33.27)	1.06 (0.83, 1.37)	0.90 (0.66, 1.22)
Ever tried other tobacco products ^1^			
No	2803 (79.83)	–	–
Yes	698 (20.17)	2.98 (2.20, 3.86)	1.66 (1.28, 2.15)
Ever tried smoking Shisha ^1^			
No	2787 (76.86)	–	–
Yes	806 (23.14)	1.99 (1.48, 2.68)	1.59 (1.20, 2.12)
Ever tried smokeless tobacco products ^1^			
No	3323 (94.57)	–	–
Yes	208 (5.43)	1.46 (0.91, 2.34)	0.69 (0.42, 1.13)
Ever tried e-cigarettes ^1^			
No	1776 (51.35)	–	–
Yes	1827 (48.65)	1.92 (1.47, 2.51)	1.67 (1.27, 2.20)

^1^Adjusted for age and sex.

#### Sex

Ever smoked sample comprised 15.14% female and 84.86% male participants. Males were significantly more likely to be in both the contemplation (RRR: 3.77, 95% CI: 1.72-8.26) and action phases (RRR: 3.17, 95% CI: 2.16-4.66) compared to females.

#### Age at cigarette Initiation

Adolescents who initiated smoking between ages 10-13 and after 13 were more likely to be in the contemplation phase rather than in maintenance phase with RRRs of 1.41 (95% CI: 1.01, 1.97) and 4.63 (95% CI: 3.06, 7.01), respectively. Similarly, for the action phase, these groups showed increased likelihoods with RRRs of 2.13 (95% CI: 1.40, 3.25) and 2.97 (95% CI: 1.91, 4.62), respectively.

#### Pocket money

Adolescents with different levels of pocket money did not show significant differences in being in the contemplation or action phases when compared to those receiving less than 20,000 rupiahs.

*Usage of Other Tobacco Products*: Adolescents who had tried other tobacco products were significantly more likely to be in both the contemplation (RRR: 2.98, 95% CI: 2.20, 3.86) and action phases (RRR: 1.66, 95% CI: 1.28, 2.15) rather than in maintenance phase.

#### Shisha, smokeless tobacco, and E-cigarette

Similar trends to other tobacco products were observed for adolescents who had tried smoking Shisha, with increased likelihoods of being in the contemplation (RRR: 1.99, 95% CI: 1.48, 2.68) and action phases (RRR: 1.59, 95% CI: 1.20, 2.12). However, no significant difference was found for those who tried smokeless tobacco products. For e-cigarette usage, there was an increased likelihood in both the contemplation (RRR: 1.92, 95% CI: 1.47, 2.51) and action phases (RRR: 1.67, 95% CI: 1.27, 2.20) as compared to maintenance phase.

### Environmental factors influencing smoking cessation behavior

**Table 2** illustrates the association between environmental factors and smoking cessation behaviors among school-going Indonesian adolescents in grades 7-12th. The significant factors are listed below.

**Table 2:** Environmental factors that influence the behavioral changes in smoking cessation among school-going Indonesian adolescents in grades 7-12, GYTS 2019.

		Contemplation vs. maintenance	Action vs. maintenance
	N (%)	RRR (95%CI) ^1^	RRR (95%CI) ^1^
Parental smoking			
No	1773 (52.41)	-	-
Yes	1628 (47.59)	1.50 (1.12, 2.01)	1.01 (0.78, 1.31)
Ever witnessed a teacher smoking in school			
No	1011 (34.38)	-	-
Yes	2044 (65.62)	1.70 (1.22, 2.36)	1.44 (1.04, 1.97)
Witness someone smoking inside the school in the last 30 days			
No	2294 (62.95)	-	-
Yes	1286 (37.05)	1.43 (1.13, 1.82)	1.31 (1.01, 1.71)
Have seen tobacco smoking on TV or in movies in last 30 days			
No	1223 (40.21)	-	-
Yes	1878 (59.79)	1.02 (0.79, 1.32)	0.85 (0.68, 1.06)
Exposure to second-hand smoke inside home in last 7 days			
No	1072 (29.06)	-	-
Yes	2529 (70.94)	1.87 (1.44, 2.43)	1.92 (1.37, 2.68)
Exposure to second-hand smoke indoors in public places in last 7 days			
No	771 (21.53)	-	-
Yes	2828 (78.47)	1.90 (1.31, 2.76)	1.33 (1.00, 1.77)
Exposure to second-hand smoke outdoors in public places in last 7 days			
No	741 (20.83)	-	-
Yes	2856 (79.17)	1.87 (1.33, 2.63)	1.30 (0.94, 1.78)
Have seen or heard anti-cigarette messages on social media in last 30 days			
No	860 (24.01)	-	-
Yes	2727 (75.99)	1.09 (0.78, 1.51)	0.92 (0.70, 1.21)
Have seen or heard anti-cigarette messages on social events in last 30 days			
No	1030 (44.85)	-	-
Yes	1218 (55.15)	0.92 (0.71, 1.20)	1.24 (0.95, 1.61)
Have seen cigarette ads on TV in last 30 days			
No	725 (23.55)	-	-
Yes	2429 (76.45)	1.42 (1.06, 1.91)	1.35 (1.09, 1.67)
Have seen cigarette ads on social media in last 30 days			
No	1639 (54.47)	-	-
Yes	1435 (45.53)	1.39 (1.10, 1.75)	1.43 (1.15, 1.77)
Have seen cigarette ads at social events in last 30 days			
No	1389 (65.94)	-	-
Yes	705 (34.06)	1.68 (1.26, 2.24)	1.61 (1.19, 2.17)
Have seen cigarette ads in sales centers in last 30 days			
No	950 (30.88)	-	-
Yes	2129 (69.12)	1.37 (0.98, 1.90)	1.04 (0.80, 1.35)
Has got free/discounted cigarettes from cigarette companies			
No	3187 (88.99)	-	-
Yes	401 (11.01)	1.64 (1.16, 2.32)	1.41 (0.97, 2.05)
Availability of cigarettes near school			
No	1735 (67.48)	-	-
Yes	870 (32.52)	2.48 (1.93, 3.19)	1.21 (0.93, 1.59)

^1^Adjusted for age and sex.

#### Parental smoking

Parental smoking showed a significant association with the contemplation phase of smoking cessation. Adolescents with parents who smoke were 1.5 times more likely to be in the contemplation phase rather than in maintenance phase (RRR = 1.50, 95% CI: 1.12 – 2.01). However, there was no significant association between parental smoking and being in the action phase of smoking cessation (RRR = 1.01, 95% CI: 0.78 – 1.31).

#### School environment

Adolescents who had witnessed teachers smoking in school were 1.7 times more likely to be in the contemplation phase (RRR = 1.70, 95% CI: 1.22 – 2.36) and 1.44 times more likely to be in the action phase (RRR = 1.44, 95% CI: 1.04 – 1.97) compared to those who had not. Similarly, witnessing someone smoking inside the school in the last 30 days was associated with an increased likelihood of being in both the contemplation (RRR = 1.43, 95% CI: 1.13 – 1.82) and action phases (RRR = 1.31, 95% CI: 1.01 – 1.71) rather than in maintenance phase.

#### Second hand tobacco exposure

Exposure to different second-hand smoke showed varying degrees of influence. Notably, exposure to second-hand smoke inside the home and in public places was significantly associated with being in both the contemplation and action phases. Specifically, exposure inside the home increased the likelihood of being in the contemplation phase by 87% (RRR = 1.87, 95% CI: 1.44 – 2.43) and the action phase by 92% (RRR = 1.92, 95% CI: 1.37 – 2.68).

#### Advertising and promotions

Exposure to cigarette ads on TV, social media, at social events, and in sale centers, as well as receiving free or discounted cigarettes from cigarette companies, were all significantly associated with an increased likelihood of being in both the contemplation and action phases of smoking cessation. The strongest association was observed for the availability of cigarettes near schools, with adolescents in this category being 2.48 times more likely to be in the contemplation phase (RRR = 2.48, 95% CI: 1.93 – 3.19).

#### Anti-cigarette messages

Exposure to anti-cigarette messages on social media or at social events did not show a significant association with being in either the contemplation or action phases.

### Knowledge and attitude and their relation with smoking cessation behavior

**Table 3** depicts the distribution and association of selected knowledge and attitude factors with smoking cessation behaviors among school-going Indonesian adolescents in grades 7-12th. The significant factors are listed below.

**Table 3:** Knowledge and attitude toward smoking and their association with behavioral changes in smoking cessation among school-going Indonesian adolescents in grades 7-12, GYTS 2019.

		Contemplation vs. maintenance	Action vs. maintenance
	N (%)	RRR (95%CI)^1^	RRR (95%CI)^1^
Awareness of cigarette smoke harm ^a^			
No	367 (10.46)	-	-
Yes	3220 (89.54)	0.65 (0.44, 0.95)	0.94 (0.68, 1.31)
Awareness of second-hand smoke harm ^b^			
No	252 (7.49)	-	-
Yes	3344 (92.51)	0.36 (0.26, 0.51)	0.61 (0.38, 0.97)
Taught about tobacco harms in school in last year			
No	903 (30.63)	-	-
Yes	2115 (69.37)	1.22 (0.92, 1.61)	1.24 (0.92, 1.67)
Cigarette smoking is joyful			
No	2519 (75.17)	-	-
Yes	810 (24.83)	7.97 (5.90, 10.77)	2.87 (2.18, 3.79)
Willingness to use a cigarette if offered by a friend			
No	2642 (73.72)	-	-
Yes	957 (26.28)	15.65 (11.65, 21.02)	5.51 (3.85, 7.88)
Support for smoking bans in indoor public places			
No	449 (12.83)	-	-
Yes	3133 (87.17)	0.69 (0.45,1.04)	0.74 (0.53, 1.03)
Support for smoking bans in outdoor public places			
No	919 (26.09)	-	-
Yes	2673 (73.91)	0.38 (0.28, 0.52)	0.68 (0.50, 0.91)

^1^ Adjusted for age and sex.

#### Awareness of cigarette smoke harm

A majority of the adolescents who ever smoked were aware of the harms of cigarette smoke (89.54%). The RRR showed that those with awareness were significantly less likely to be in the contemplation phase rather than in maintenance phase of smoking cessation (RRR = 0.65, 95% CI: 0.44, 0.95). However, there was no significant association between awareness of cigarette smoke harm and being in the action phase of smoking cessation (RRR = 0.94, 95% CI: 0.68, 1.31).

*Awareness of second-hand smoke harm*: A significant majority of adolescents who ever smoked (92.51%) were aware of the harms associated with second-hand smoke. Those aware were significantly less likely to be in both the contemplation (RRR = 0.36, 95% CI: 0.26, 0.51) and action phases (RRR = 0.61, 95% CI: 0.38, 0.97) rather than in maintenance phase.

#### Taught about tobacco harms in school in last year

About 69.37% of the adolescents who ever smoked reported being taught about the harms of tobacco in school in the last year. This educational exposure did not show a significant association with being in either the contemplation (RRR = 1.22, 95% CI: 0.92, 1.61) or action phases (RRR = 1.24, 95% CI: 0.92, 1.67).

#### Cigarette smoking is joyful

A subset of adolescents who ever smoked (24.83%) believed that cigarette smoking is enjoyable. This belief was strongly associated with higher odds of being in both the contemplation (RRR = 7.97, 95% CI: 5.90, 10.77) and action phases (RRR = 2.87, 95% CI: 2.18, 3.79) of smoking cessation, as compared to the maintenance phase.

#### Willingness to use a cigarette if offered by a friend

About 26.28% of adolescents reported they would use a cigarette if offered by a friend, which was strongly associated with increased likelihood of being in both the contemplation (RRR = 15.65, 95% CI: 11.65, 21.02) and action phases (RRR = 5.51, 95% CI: 3.85, 7.88).

#### Support for smoking bans in Indoor public places

While a substantial proportion of adolescents (87.17%) were in favor of banning smoking in indoor public places, this attitude was not significantly associated with being in the contemplation (RRR = 0.69, 95% CI: 0.45, 1.04) or action phases (RRR = 0.74, 95% CI: 0.53, 1.03).

#### Support for smoking bans in outdoor public places

Similarly, a significant majority (73.91%) supported banning smoking in outdoor public places. This support was associated with a reduced likelihood of being in the contemplation phase (RRR = 0.38, 95% CI: 0.28, 0.52), as well as the action phase (RRR = 0.68, 95% CI: 0.50, 0.91), compared to the maintenance phase.

## Discussion

Leveraging the Transtheoretical Model of Behavioral Change (TTM), this study provides insights into the adolescents’ journey towards smoking cessation, understanding the association of demographic factors, exposure to anti-cigarette messages, and participants’ knowledge and attitudes with being at each stage of the process. Our results highlight a significant relationship between age and smoking cessation behaviors. Adolescents aged ≥14 years demonstrated a higher likelihood of being in the contemplation phase as compared to maintenance phase, consistent with prior research indicating that age plays a critical role in smoking behaviors (12, 13). Additionally, early cigarette initiation was associated with an increased likelihood of being in the contemplation and action phases. It has been shown that onset of regular smoking at an early age is associated with higher odds of nicotine dependence and lower odds of attempting and intending to quit. Although our findings cannot predict the future changes in smoking behaviors due to its cross-sectional design, it may suggest that efforts to prevent access to tobacco products at an early age could reduce nicotine addiction and promote cessation later in life (14, 15). The findings suggested that male adolescents are more likely to be in both the contemplation and action phases as compared to maintenance phase. Similarly, prior research on smoking cessation among Indonesian adolescents has noted a greater tendency toward quitting smoking and maintaining a free smoke life among females (16, 17). In Indonesia, Indonesian men rank first in countries with a prevalence of smokers in the world at 63%, while only 5% of Indonesian women are smokers, making smoking the majority behavior of Indonesian men (18). Cultural factors, peer influences, and societal norms around masculinity and smoking in Indonesia may contribute to this discrepancy (19), and interventions tailored to address these gender-specific factors could be beneficial.

In this study, parental smoking was identified as a significant environmental factor associated with smoking cessation behaviors among Indonesian adolescents. The findings showed that adolescents with smoking parents were more likely to be in the contemplation phase, aligning with previous research that highlights the influence of parental behaviors on adolescent smoking (20, 21). Although the adolescents in this study were not followed up to understand the progress or relapse in relation to parental smoking, prior studies have shown that parental smoking predicts future initiation and regular smoking among adolescents, suggesting intergenerational transmission of smoking behavior within families (20). Another important environmental factor that increases the likelihood of being in the contemplation phase is witnessing a teacher smoking at school. Evidence indicate that it could encourage cigarette initiation among students (22, 23), and studies have suggested the need to strengthen pre-service and in-service teacher training programs in smoking education, make smoking cessation programs available to teachers who want to stop smoking, and implement smoke-free policies in schools (24, 25).

Our study adds to the growing body of literature on the influence of using other tobacco products and exposure to smoke on smoking cessation behaviors (26–28). Adolescents who tried other tobacco products or were exposed to second-hand smoke were more likely to be in the contemplation and action phases rather than in maintenance phase. Although the cross-sectional design of this study hinders the predication of future progress or relapse among study population, this finding suggest the importance of comprehensive tobacco control policies and educational programs that address all forms of tobacco use and exposure. A smoke-free environment has been shown to support and encourage cessation decisions and attempts among smokers trying to quit (27). Smoke-free workplaces and communities make youth and young adults less likely to start smoking due to several factors, including lower visibility of people who smoke, fewer opportunities to smoke alone or with others, and reduced social acceptability for smoking (28). Additionally, the findings of this study indicated higher experiences of e-cigarette smoking among those in contemplation and action phases as compared to those in maintained phase. Studies on e-cigarette use and smoking cessation indicated inconsistent results among the adolescent population (29). Although e-cigarette use could be beneficial in smoking cessation in adult population, it could be a risk factor for smoking initiation among adolescents (29). Further, longitudinal studies are suggested to disentangle the complex interaction between smoking cessation attempts and the e-cigarette effect among adolescent population.

The results of the study among Indonesian adolescents suggest that exposure to cigarette ads on TV, social media, at social events, and in sale centers, as well as receiving free or discounted cigarettes from cigarette companies, were all significantly associated with an increased likelihood of being in both the contemplation and action phases of smoking cessation. These findings are consistent with previous research that has highlighted the influence of cigarette advertising and promotion on youth smoking behavior in Indonesia (30, 31) in which the cigarette ads were shown to be encouraging youths to initiate and maintain a positive attitude toward smoking (30) which hinder the attempts to stop or maintain smoke-free lifestyle. Moreover, anti-cigarette messages appeared to be ineffective in encouraging adolescents in quitting and maintain a smoke-free life which was consistent with a recent study in Indonesia (9). These findings have important implications for tobacco control policies and educational programs in Indonesia where tobacco consumption has limited control, regardless of age or sex (32). Additionally, a higher availability of cigarettes near schools appeared to be associated with adolescents being in the contemplation phase. The findings concerning the availability of cigarettes near schools and adolescent smoking are inconsistent. While some studies suggest that the density/proximity of tobacco outlets around schools shows no or inverse association with adolescent smoking (33), other studies suggest that the availability of cigarettes near schools is the strongest environmental factor associated with smoking cessation behaviors among adolescents (34, 35). Therefore, comprehensive tobacco control policies that address all forms of tobacco use and exposure, including cigarette advertising, promotion, and sale could be crucial components of strategies to promote smoking cessation among adolescents.

With regards to knowledge and attitude toward smoking, this study indicated that awareness of the harms of cigarette and second-hand smoke was associated with a reduced likelihood of being in the contemplation and action phases, emphasizing the role of knowledge in smoking cessation. In fact, previous studies have shown that knowledge about the harms of smoking is a key element for smoking cessation and prevention (36, 37). The knowledge-attitude-practice model suggests that behavior change involves acquiring relevant knowledge, changing related attitudes, and altering practices (36). However, on the other hand, the belief that cigarette smoking is enjoyable and willingness to use a cigarette if offered by a friend were strongly associated with an increased likelihood of being in both phases. Studies have shown that social cues, such as smoking by peers and family members, are strong predictors of smoking initiation and maintenance among youth (38). The belief that smoking is enjoyable and willingness to use a cigarette if offered by a friend are attitudes that are often shaped by social cues (39). Interventions that target social norms and attitudes towards smoking have been found to be effective in reducing smoking initiation and increasing smoking cessation among youth (39, 40). This highlights a potential area for targeted interventions, focusing on changing attitudes and beliefs around smoking enjoyment and social smoking.

## Limitations and future research

This study is not without its limitations. While our study provides valuable insights into smoking cessation behaviors among adolescents, it is essential to acknowledge the limitation inherent in our cross-sectional design. The comparison between action and maintenance stages lacks temporal context, as our data do not capture the longitudinal trajectory of smoking cessation. Thus, our findings may reflect associations rather than causality, and caution should be exercised in interpreting the results. Longitudinal studies are warranted to elucidate the dynamic nature of smoking cessation behaviors over time, providing a more comprehensive understanding of the factors influencing sustained cessation. Additionally, since the Indonesian GYTS 2019 was conducted in school, the results are not representative of adolescents outside the school who might be at a higher risk of risky addictive behaviors (9, 41). Additionally, we couldn’t determine the number of individuals in the pre-contemplation phase due to data constraints. A lower response rate to questions on cessation intention and timing compared to total smokers suggests potential under-reporting or temporal smoking behavior. Although we adjusted for age and sex, residual confounding factors may exist, impacting our findings. Despite limitations, our study provides valuable insights into adolescent smoking cessation, emphasizing the need for targeted interventions and further research. Another limitation of our study is the discrepancy in timing observed during the assessment of smoking cessation stages. While the TTM traditionally categorizes individuals based on a 6-month timeframe for cessation, the GYTS questionnaire utilized a 3-month timeframe. Consequently, we redefined the action stage as individuals who had ceased smoking for the last 3 months and the maintenance stage as those who had abstained from smoking for more than 3 months. This deviation may limit comparability with studies adhering strictly to the traditional TTM categorization. To address this, future iterations of the GYTS questionnaire should consider aligning the timing of smoking cessation questions with established models like the TTM to enhance consistency and comparability across studies.

## Conclusion

This study provides valuable insights into the factors associated with smoking cessation behaviors among school-going Indonesian adolescents. Tailored interventions that address age-specific barriers, gender disparities, cultural influences, environmental factors, and knowledge and attitudes toward smoking are crucial. Strengthening tobacco control policies, particularly in schools and public places, could further support adolescent smoking cessation efforts. Moreover, there is a need for longitudinal studies to examine the evolving nature of smoking cessation behaviors over time, facilitating a more comprehensive understanding of the factors that contribute to sustained cessation.

## Data availability statement

The GYTS datasets are publicly available data and are available in the CDC website repository through the following link: https://extranet.who.int/ncdsmicrodata/index.php/catalog/926.

## Ethics statement

This was a secondary analysis of the Indonesia Global Youth Tobacco Survey in 2019 (GYTS2019). The GYTS2019 protocol received approval and guidance from the Indonesia Health Research Ethics Commission and, National Health Research and Development Agency. Students were informed about the survey objectives, data confidentiality, and assurances that non-participation wouldn’t affect their grades before the survey. All students and guardians were then provided with a consent form before the survey.

## Author contributions

OD: Conceptualization, Data curation, Formal analysis, Investigation, Methodology, Validation, Visualization, Writing – original draft, Writing – review & editing.
