# Supplementary material for: Predictor of smoking cessation among school-going adolescents in Indonesia: a secondary analysis based on the transtheoretical model of behavioral change
Source: Front Psychiatry. 2024 Mar 7;15:1374731. doi: 10.3389/fpsyt.2024.1374731 (PMC10954880; doi:10.3389/fpsyt.2024.1374731)
Supplement: Supplementary file 1 [file Table_1.docx]

| Appendix 1. The selected variables and corresponding questions and alternative responses; Indonesia GYTS 2019 | | | |
| --- | --- | --- | --- |
|  | Variable | Questions | Answers |
| **Individual factors** | Age at cigarette initiation | How old were you when you first tried a cigarette? | 1. I have never tried smoking a cigarette  2. 7 years old or younger  3. 8 or 9 years old  4. 10 or 11 years old  5. 12 or 13 years old  6 .14 or 15 years old  7. 16 years old or older |
|  | Pocket money (rupiah) | During an average week, how much money do you have that you can spend on yourself, however you want? | 1. I usually don't have any spending money  2 Less than Rp. 11,000  3 Rp. 11,000 - Rp. 20,000  4. Rp. 21,000 - Rp. 30,000  5. Rp. 31,000 - Rp. 40,000  6. Rp. 41,000 - Rp. 50,000  7. More than Rp. 50,000 |
|  | Ever tried other tobacco products ^1^ | Have you ever tried or experimented with any form of smoked tobacco products other than cigarettes (such as klobot, klembak menyan, hand-rolled tobacco/tingwe, shisha)? | 1. Yes  2. No |
|  | Ever tried smoking Shisha | Have you ever tried or experimented with shisha smoking, even one or two puffs? | 1. Yes  2. No |
|  | Ever tried smokeless tobacco products | Have you ever tried or experimented with any form of smokeless tobacco products (such as  chewing tobacco, betel leaf with cane, and betel nut with tobacco)? | 1. Yes  2. No |
|  | Ever tried e-cigarettes | Before today, had you ever heard of electronic cigarettes or e-cigarettes? | 1. Yes  2. No |
| **Environmental factors** | Parental smoking | Do your parents smoke tobacco? | 1. None  2. Father only  3. Mother only  4. Both  5. Don't know |
|  | Ever witnessed a teacher smoking in school | During school hours, how often do you see teachers smoking in the school building (such as in the classroom, in the teacher's room, toilets, or other rooms in the building)? | 1. About every day  2. Sometimes  3. Never  4. Don't know |
|  | Witness someone smoking inside the school in the last 30 days | During the past 30 days, did you see anyone smoke inside the school building or outside on school property? | 1. Yes  2. No |
|  | Have seen tobacco smoking on TV or in movies in last 30 days | During the past 30 days, did you see any people using tobacco on TV, in videos, or in movies? | 1. I did not watch TV in the past 30 days  2. Yes  3. No |
|  | Exposure to second-hand smoke inside home in last 7 days | During the past 7 days, on how many days has anyone smoked inside your home, in your presence? | 1. 0 days  2. 1 to 2 days  3. 3 to 4 days  4. 5 to 6 days  5. 7 days |
|  | Exposure to second-hand smoke indoors in public places in last 7 days | During the past 7 days, on how many days has anyone smoked in your presence, inside any enclosed public place, other than your home (such as schools, malls, restaurants, shopping centers, theaters, cafes, health service facilities, public transportation, indoor sports venues)? | 1. 0 days  2. 1 to 2 days  3. 3 to 4 days  4. 5 to 6 days  5. 7 days |
|  | Exposure to second-hand smoke outdoors in public places in last 7 days | During the past 7 days, on how many days has anyone smoked in your presence, at any outdoor public place (such as playgrounds, curbside, building entrances, parks, beaches, sports fields)? | 1. 0 days  2. 1 to 2 days  3. 3 to 4 days  4. 5 to 6 days  5. 7 days |
|  | Have seen or heard anti-cigarette messages on social media in last 30 days | During the past 30 days, did you see or hear any anti-cigarette media messages on television, radio, internet, billboards, posters, newspapers, magazines, or movies? | 1. Yes  2. No |
|  | Have seen or heard anti-cigarette messages on social events in last 30 days | uring the past 30 days, did you see or hear any anti-cigarette messages at sports events, fairs, concerts, or community events, or social gatherings? | 1. I did not go to sports events, fairs, concerts, or community events, or social gatherings in the past 30 days?  2. Yes  3. No |
|  | Have seen cigarette ads on TV in last 30 days | Over the past 30 days, have you seen cigarette advertisements/promotions/brand names/cigarette logos on television? | 1. I did not watch TV in the past 30 days  2. Yes  3. No |
|  | Have seen cigarette ads on social media in last 30 days | During the past 30 days, did you see any cigarette advertisements when you opened in the Internet or social media? | 1. I did not open the Internet or social media in the past 30 days  2. Yes  3. No |
|  | Have seen cigarette ads at social events in last 30 days | During the past 30 days, did you see any advertisements for cigarette products at community events/social gatherings? | 1. I did not attend any community events/social gatherings in the past 30 days  2. Yes  3. No |
|  | Have seen cigarette ads in sales centers in last 30 days | During the past 30 days, did you see any advertisements or promotions for cigarette products in sales centers (such as shops, stalls, kiosks and minimarket)? | 1. I haven't visited the sales centers in the past 30 days  2. Yes  3. No |
|  | Has got free/discounted cigarettes from cigarette companies | Have you ever got free cigarettes/discounted cigarette coupons/vouchers from cigarette companies? | 1. Yes  2. No |
|  | Availability of cigarettes near school | Can you purchase cigarettes near your school? | 1 Yes  2 No |
| **knowledge and attitude** | Awareness of cigarette smoke harm | Do you think smoking cigarettes is harmful to your health? | 1. Definitely not  2. Probably not  3. Probably yes  4. Definitely yes |
|  | Awareness of second-hand smoke harm | Do you think the smoke from other people's cigarettes smoking is harmful to you? | 1. Definitely not  2. Probably not  3. Probably yes  4. Definitely yes |
|  | Taught about tobacco harms in school in last year | During the past 12 months, were you taught in any of your classes about the dangers of tobacco use? | 1. Yes  2. No  3. I don't know |
|  | Cigarette smoking is joyful | Do you agree or disagree with the following: "I think I might enjoy smoking a cigarette." | 1. I currently smoke cigarettes  2. Strongly agree  3. Agree  4. Disagree  5. Strongly disagree |
|  | Willingness to use a cigarette if offered by a friend | If one of your best friends offered you a cigarette product, would you use it? | 1. Definitely not  2. Probably not  3. Probably yes  4. Definitely yes |
|  | Support for smoking bans in indoor public places | Are you in favor of banning smoking inside enclosed public places (such as in health facilities, public transportation, teaching and learning places, shops, restaurants, shopping centers, theaters, cafes, indoor sports clubs)? | 1 Yes  2 No |
|  | Support for smoking bans in outdoor public places | Are you in favor of banning smoking at outdoor public places (such as playgrounds, roadside, building entrances, parks, on the beach, on sports fields)? | 1 Yes  2 No |
